# Subcutaneous Phaeohyphomycosis Cyst Associated with *Medicopsis romeroi* in an Immunocompromised Host

**DOI:** 10.1007/s11046-016-0017-4

**Published:** 2016-05-18

**Authors:** Alireza Abdolrasouli, Ximena Gonzalo, Anita Jatan, Gordon J. McArthur, Nicholas Francis, Berge S. Azadian, Andrew M. Borman, Elizabeth M. Johnson

**Affiliations:** 1 Department of Medical Microbiology, 4th Floor East Wing Laboratory Block, Charing Cross Hospital, Imperial College Healthcare NHS Trust, Fulham Palace Road, London, W6 8RF UK; 2 Fungal Pathogens Immunobiology Laboratory, National Heart and Lung Institute, Imperial College London, London, UK; 3 Department of Pathology, Charing Cross Hospital, Imperial College Healthcare NHS Trust, London, UK; 4 Department of Plastic Surgery, Chelsea and Westminster Hospital, Chelsea and Westminster NHS Foundation Trust, London, UK; 5 National Mycology Reference Laboratory and National Collection of Pathogenic Fungi, Public Health England, Bristol, UK

**Keywords:** *Medicopsis romeroi*, Phaeohyphomycoses, Mycoses, Antifungal susceptibility testing, Subcutaneous cyst

## Abstract

An 88-year-old man, receiving prednisolone for sarcoidosis, presented with a discrete keratotic lesion on the dorsum of his right hand following the placement of an intravenous cannula a month prior to its appearance. *Medicopsis romeroi* was isolated from the tissue and identified by sequencing the internal transcribed spacer region ITS-1 and the D1-2 fragment of the 28S rDNA gene. Histopathological examination showed fungal hyphae in the internal inflammatory cells layer and within the histocyte-macrophage layer, highly suggestive of deep mycosis. The patient was successfully treated with surgical excision of the cyst. *M. romeroi* exhibited high MIC values for echinocandin drugs in vitro, but appeared susceptible to newer triazole agents, amphotericin B and terbinafine. This is the first report of a subcutaneous phaeohyphomycotic cyst occurring following the placement of an intravenous cannula. This report highlights the potential role of *M. romeroi* as an emerging cause of deep, non-mycetomatous infection in immunocompromised patients.

## Introduction

Phaeohyphomycoses are fungal infections of the skin, or internal organs, caused by darkly pigmented, melanized fungi, which are widely distributed in the environment [1]. In recent years, the incidence of phaeohyphomycosis as well as the diversity of causative organisms has been reported to be increasing globally [2]. Clinically, they are involved in diseases ranging from mild, superficial infections [3] to fatal cerebral phaeohyphomycosis in otherwise healthy individuals [4].

Subcutaneous phaeohyphomycoses is an uncommon localised fungal infection of the deep dermis and subcutaneous tissues caused by a heterogeneous group of dematiaceous fungi [5]. Infection is thought to result from traumatic implantation of the causative fungal organism into the subcutaneous tissue. This form of infection is more common in warm climates and has been reported mainly in immunocompromised hosts [6]. It commonly presents as a single, well-encapsulated, subcutaneous mass or a nodule at the site of previous trauma, commonly on the extremities. The common causative organisms reported include *Exophiala*, *Alternaria*, *Phialophora*, *Cladophialophora*, and *Curvularia/Bipolaris* species [5, 7]; however, many others have been implicated on occasion [8, 9].

*Medicopsis romeroi*, formerly known as *Pyrenochaeta romeroi*, is a rare agent of human black-grain eumycetoma [10, 11]. Recently *M. romeroi* emerged as a cause of deep, non-mycetomatous infections mainly in immunocompromised patients [12–19]. We report here a case of subcutaneous phaeohyphomycotic cyst of the hand caused by *M. romeroi* in an immunocompromised man.

### Case Report

In July 2015, an 88-year-old British man of mixed heritage (Caucasian and Afro-Caribbean) but resident in England most of his life, presented with a 5-month history of a discrete keratotic lesion on the dorsum of his right hand, following the placement of an intravenous cannula a month prior to its appearance. His medical history included chronic obstructive pulmonary disease, gout, benign prostatic hyperplasia, deep venous thrombosis, sarcoidosis and linear IgA dermatosis. He was on multiple medications for his comorbidities, among which was the immunosuppressant prednisolone. He was treated for leprosy and Bell’s palsy in 1994 and denied any recent overseas travel.

Examination confirmed a necrotic lesion 20 × 12 mm with a 3 × 3 mm well-circumscribed whitened area. The lesion was confined to the skin and subcutaneous tissue with no extension to extensor tendons, bone, lymph node or neurovascular involvement. Cellulitis, tracking lymphangitis and regional lymphadenopathy were absent.

**Fig. 1:**
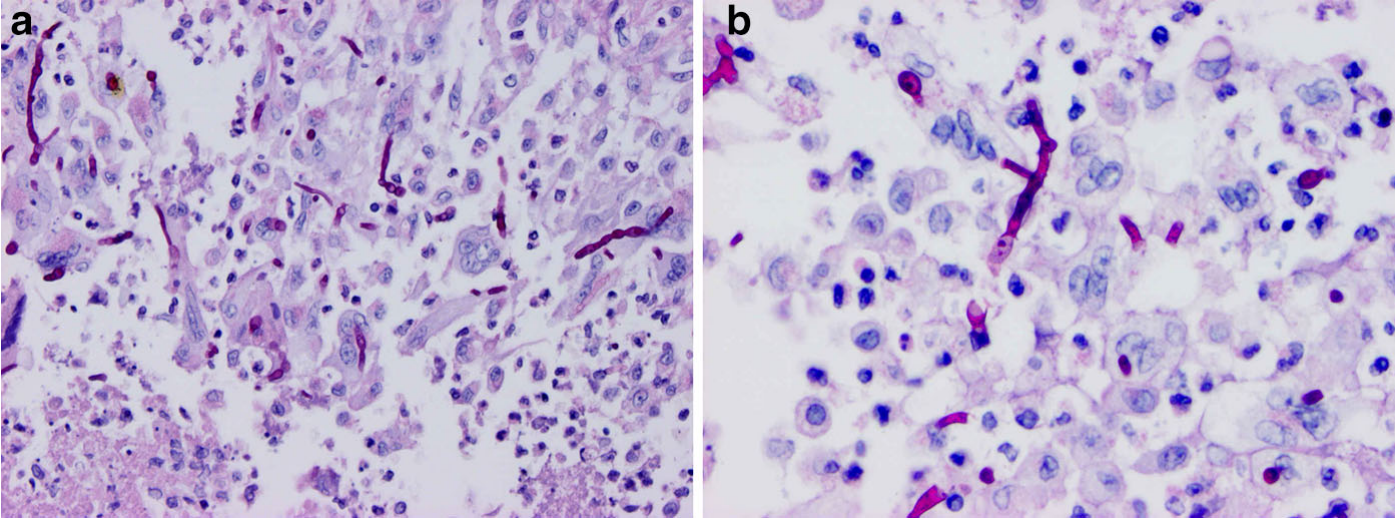
Fungal hyphae in the internal inflammatory cells layer and within the histocyte-macrophage layer, PAS stain with magnification of ×200 (**a**) and ×400 (**b**)

**Fig. 2:**
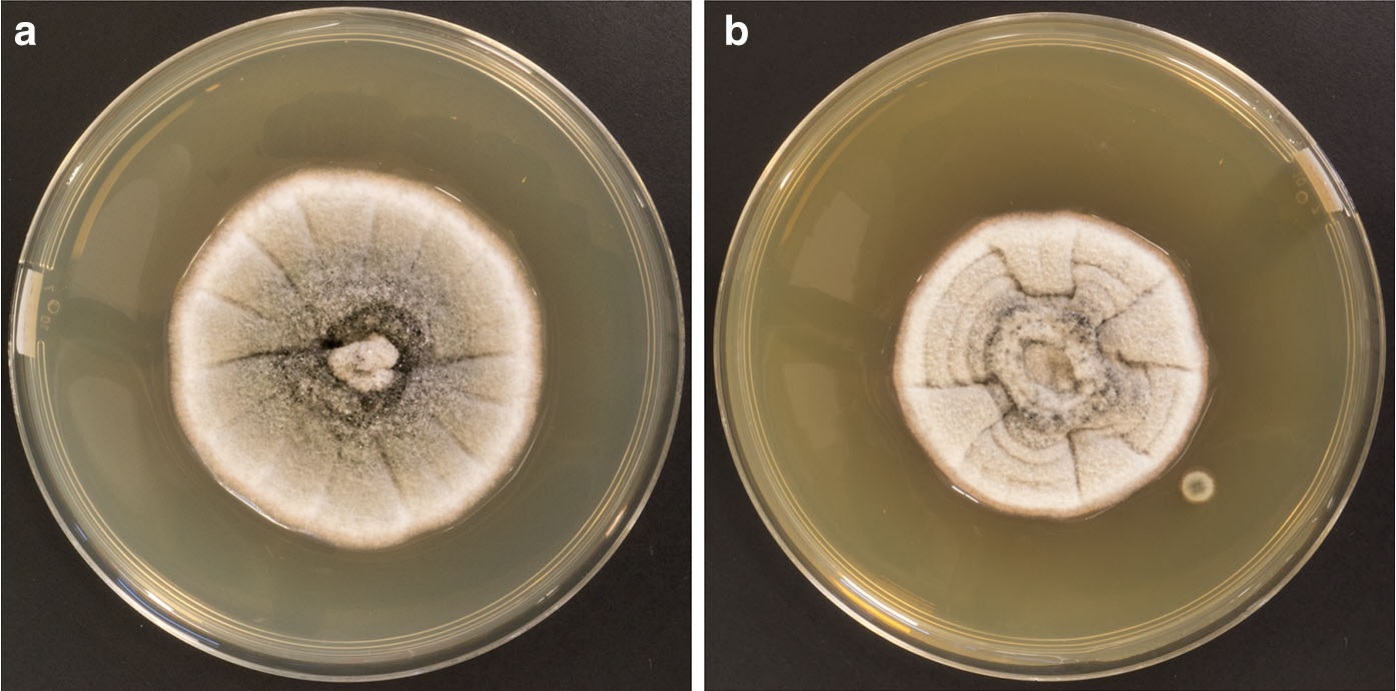
Colonies of *M. romeroi* on SDA (**a**), and malt extract agar (**b**) after 10 days incubation at 28–30 °C in *dark*. Colonies were initially *brownish* but became *olivaceous grey* and *floccose* with age on both culture media

**Fig. 3:**
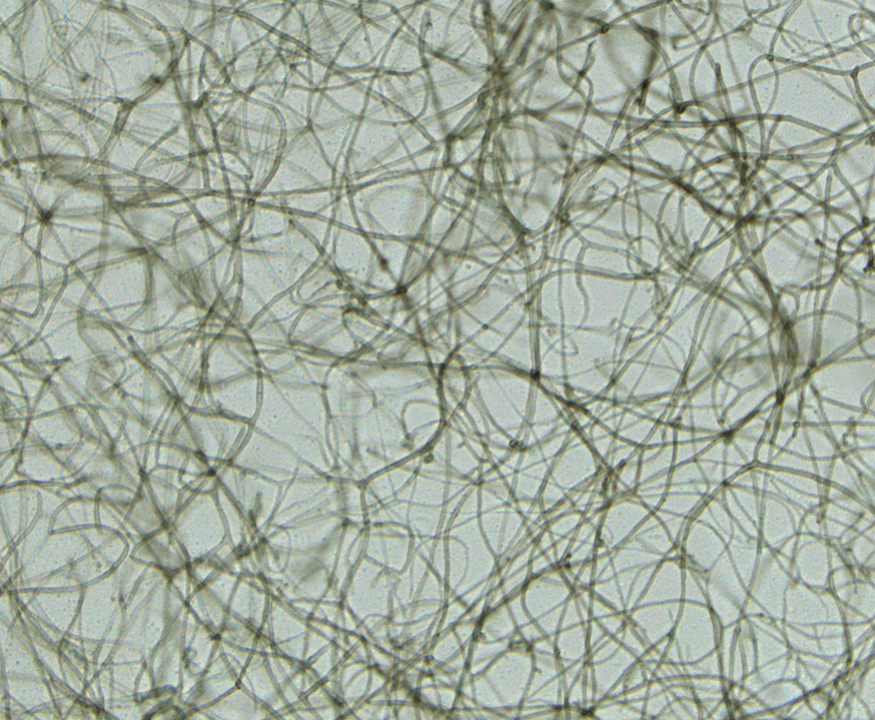
*Broad*, *septate*, *branched* and *dark brown hyphae* consistent with dematiaceous fungi. Magnification ×400

With a differential diagnosis of atypical abscess and epidermal inclusion cyst, the cyst was removed by elliptical excision to subcutis with primary closure; the patient received co-amoxiclav and co-codamol upon discharge. In 10 days of follow up, there was no sign of infection. Histological examination showed an abscess with central neutrophils and prominent surrounding infiltrate of epithelioid macrophages with some giant cells. Periodic acid-Schiff stain showed fungal hyphae in the internal inflammatory cells layer and within the histocyte-macrophage layer (Fig. 1), highly suggestive of deep mycosis. Microscopic examination of the tissue sample with calcofluor white revealed fungal elements, but no granules were observed macroscopically. A dematiaceous mould grew after 10 days of incubation on Sabouraud dextrose agar at both 30 and 37 °C (Fig. 2). Microscopic examination of the culture showed broad, septate, branched and dark brown hyphae consistent with dematiaceous fungi. No conidia were observed (Fig. 3). With the diagnosis of phaeohyphomycotic cyst, and in view of our patient’s immunocompromised state, he was contacted to offer antifungal therapy, which was refused.

For species identification, the internal transcribed spacer region ITS-1 and the D1-2 fragment of the 28S rDNA gene were amplified by PCR using extracted DNA and panfungal primers using previously established protocols [20, 21]. The sequences of the resulting amplicons (European Molecular Biology Laboratory (EMBL) accession nos. LN999520 and LN999521) were both 100 % identical to reference sequences of *M. romeroi* present in public sequence databases, and to the type strain of *M. romeroi* in the National Collection of Pathogenic Fungi (NCPF 2301) housed at the National Mycology Reference Laboratory, Public Health England, Bristol, UK (data not shown). The isolate from the current case has been preserved in inert form in the NCPF with the unique identifier NCPF 7881.

Antifungal susceptibility testing was performed by microbroth dilution according to the Clinical and Laboratory Standards Institute (CLSI) M38-A2 guideline [22]. The minimum inhibitory concentrations (MICs) of antifungal drugs were: amphotericin B (0.25 μg/ml), fluconazole (>64 μg/ml), itraconazole (0.5 μg/ml), voriconazole (0.5 μg/ml), posaconazole (0.25 μg/ml) and terbinafine (0.125 μg/ml). Caspofungin, anidulafungin and micafungin all gave minimum effective concentration (MEC) of 4.0 μg/ml.

## Discussion

Phaeohyphomycosis is an uncommon fungal infection, although its incidence has been reported to be on the rise globally [1, 2]. Most phaeohyphomycosis infections are caused by *Exophiala*, *Alternaria, Cladophialophora*, *Phialophora* or *Curvularia*/*Bipolaris* species; however, many others have been implicated on occasion [5, 7]. Infection is most often acquired from traumatic implantation of the causative agent into subcutaneous tissue. The usual clinical presentation is the asymptomatic development of a single subcutaneous mass (nodule, cyst or abscess) at the site of prior trauma [6]. *M. romeroi* is a saprophytic fungus widely distributed worldwide, where it grows on soil and vegetation, particularly in tropical climates. The majority of the previously reported cases have occurred in patients with a history of residence in India, Pakistan and Venezuela [12].

Although the etiological role of *M. romeroi* in black-grain mycetoma is well established [10, 23], it has only recently been recognised as a cause of deep, non-mycetomatous infections [12–17, 19]. In contrast to mycetoma, no discharge or visible grains are present. This could potentially be a relatively new clinical condition presenting as subcutaneous nodule, cyst or abscess caused by this species mostly in immunocompromised patients. Our patient was on long-term prednisolone for sarcoidosis which we believe could potentially contribute to his recent fungal infection with *M. romeroi*. Among the immunosuppressed population, solid organ transplant recipients and patients receiving chronic corticosteroids are at risk for dematiaceous fungal infections [6, 24, 25]. Of note, most cases of subcutaneous phaeohyphomycosis caused by *M. romeri* have been reported among renal transplant recipients [15, 17, 19, 26]. Individual cases have also been described in patients with acute lymphoblastic leukaemia [13], diabetes mellitus [14] or those receiving prolonged immunosuppressant agents [16, 18]. However, cases have been described in previously healthy immunocompetent individuals [12] where cysts are often chronic and relatively asymptomatic.

Subcutaneous phaeohyphomycotic lesions are often surgically excised which is usually curative with no further antifungal therapy [12, 27]. Systemic antifungal therapy is used in patients with refractory or recurrent infections perhaps due to incomplete resection of the lesion. The agents that have most frequently been used include amphotericin B, 5-flucytosine, itraconazole and terbinafine. The newer triazoles have moderate-to-excellent in vitro antifungal activity against dematiaceous moulds and can be given safely for prolonged periods for treatment of phaeohyphumycosis [27].

Information on the antifungal susceptibility of *M. romeroi* is rather limited due to lack of high number of clinical isolates and absence of established breakpoints. In addition, relation between in vitro MIC values and clinical outcome of infection is poorly understood. The available evidence suggests considerable strain-specific variation in MIC values with respect to amphotericin B and itraconazole, elevated MICs for fluconazole, ketoconazole, and lower MICs for voriconazole [23, 28]. Our isolate demonstrated a high MIC with fluconazole; however, lower MICs were observed to the newer triazole agents. High MECs observed with the echinocandin group, in agreement with previous reports [23, 28], suggest these agents would not be a good choice for treatment of *M. romeroi* infections. Terbinafine might be efficacious against this fungus.

## Conclusion

This report highlights the potential role of *M. romeroi* as an emerging cause of subcutaneous, phaeohyphomycotic cysts in immunocompromised patients with no travel history to tropical or subtropical areas. Recognition of infections caused by dematiaceous fungi remains challenging as many isolates are difficult to identify by conventional methods and require molecular approaches.
